# A qualitative study exploring the difficulties influencing decision making at the end of life for people with dementia

**DOI:** 10.1111/hex.12593

**Published:** 2017-06-22

**Authors:** Kethakie Lamahewa, Rammya Mathew, Steve Iliffe, Jane Wilcock, Jill Manthorpe, Elizabeth L Sampson, Nathan Davies

**Affiliations:** ^1^ Research Department of Primary Care and Population Health University College London London UK; ^2^ Social Care Workforce Research Unit King's College London London UK; ^3^ Division of Psychiatry Marie Curie Palliative Care Research Department University College London London UK; ^4^ Barnet Enfield and Haringey Mental Health Trust Liaison Team North Middlesex University Hospital London UK

**Keywords:** decision‐making, dementia, end‐of‐life, family caregivers, palliative care, qualitative research

## Abstract

**Background:**

Dementia is a progressive neurodegenerative condition characterized by declining functional and cognitive abilities. The quality of end of life care for people with dementia in the UK can be poor. Several difficult decisions may arise at the end of life, relating to the care of the person with dementia, for example management of comorbidities.

**Objective:**

To explore difficulties in decision making for practitioners and family carers at the end of life for people with dementia.

**Design:**

Qualitative methodology using focus groups and semi‐structured interviews and thematic analysis methods.

**Settings and participants:**

Former (n=4) and current (n=6) family carers of people with experience of end of life care for a person with dementia were recruited from an English dementia voluntary group in 2015. A further 24 health and care professionals were purposively sampled to include a broad range of expertise and experience in dementia end of life care.

**Results:**

Four key themes were identified as follows: challenges of delivering coherent care in dynamic systems; uncertainty amongst decision makers; internal and external conflict amongst decision makers; and a lack of preparedness for the end of life. Overarching difficulties such as poor communication, uncertainty and conflict about the needs of the person with dementia as well as the decision maker's own role can characterize decision making at the end of life.

**Conclusions:**

This study suggests that decision making at the end of life for people with dementia has the potential to be improved. More planning earlier in the course of dementia with an on‐going approach to conversation may increase preparedness and family carers’ expectations of end of life.

## 1 BACKGROUND

Health and social care professionals report they lack confidence in decision making with respect to end of life care amongst people with dementia, and this is compounded by a lack of professional guidance in this area, with much of the guidance on end of life care focussing primarily on people with cancer.^1^

Dementia presents a global health challenge. Although incidence is declining,^2^ the prevalence of dementia is still rising across the world because of the ageing population.^3^, ^4^, ^5^ It is a progressive neurodegenerative condition characterized by a gradual decline in cognitive abilities and an accompanying increase in health and social care needs. Median life expectancy from the time of diagnosis is estimated to be around three and a half years.^6^ However, it is often difficult to estimate the prognosis for someone with dementia. Unlike diseases such as cancer, where the prognosis and course of illness are relatively well‐understood, the trajectory of dementia is much more uncertain and heterogeneous and can be punctuated with declines related to acute illness.^7^, ^8^ End of life in the UK is considered to be the final 12 months of life, as defined by the National End of Life Care Strategy.^9^ The end of life phase in dementia is often recognized late^10^ and therefore may not be managed optimally. In this study, we define end of life care as a period of time during which the individual or family and professionals recognize that the person is dying, which could potentially be a period of up to 1‐2 years.^11^

End of life symptoms experienced by people with dementia may not be different to those of other terminal conditions such as cancer, but may be experienced for a much longer period of time.^12^ Common clinical complications include recurrent infections (such as pneumonia), difficulties with swallowing, incontinence and pressure sores.^7^ As the person with dementia may lack capacity, difficult decisions sometimes have to be made by family members and health and care professionals about end of life care, supported by the Mental Capacity Act 2005. The Mental Capacity Act 2005 states that decisions made are in the best interest of the person and their rights are respected.^13^ These decisions may include establishing ceilings of care, for example deciding when to stop providing treatments such as antibiotics for recurrent infections, or what to do if someone is no longer able to swallow and eat food. Previous studies have identified factors such as the differing views, expectations and priorities of families and practitioners as factors which complicate the decision‐making process.^14^, ^15^ The care process has also been framed as a triad involving the person with dementia, any family carers and professionals from the health and care systems.^16^ Few clinical guidelines focus specifically on end of life for people with dementia and those that exist have been developed by organisations such as the National Council for Palliative Care and the Alzheimer's Society.^17^, ^18^, ^19^, ^20^, ^21^

In this article, we identify and explore influences on decision making for practitioners and family carers at the end of life for people with dementia.

## 2 METHODS

### 2.1 Design

A qualitative methodology was chosen for this study using semi‐structured interviews and focus groups to explore personal and professional accounts in a sensitive manner. Both interviews and focus groups were analysed using thematic analysis methods.

### 2.2 Participants and setting

Former and current family carers of a person with dementia were recruited from a national dementia voluntary sector group, purposively inviting those with experience of providing care at home or experiencing it in hospital, and those who had experience of providing end of life. Invitations were sent either in the post or by email from the voluntary sector organisation to potential participants. Interested participants returned a postal expression of interest to the research team or replied in email to the voluntary sector, and they were then followed up with a telephone call from the research team to explain the study in more detail and confirm eligibility.

We purposively sampled a broad range of professionals working with people with dementia at the end of life in England including; general practitioners, palliative care nurses and physicians, geriatricians, speech and language therapists, hospital nurses, health‐care assistants, community nurses and pharmacists. Professionals were recruited through the Dementias and Neurodegeneration (DeNDRoN) coordinating centre and the Comprehensive Local Research Network (CLRN). Finally, the research team invited experts within the field of dementia and end of life care to take part in the study, using snowballing methods to complete recruitment.^22^ Invitations for professionals were sent via email from the research team or DeNDRoN and asked to reply via email or telephone to the research team.

### 2.3 Procedure

One focus group (n=3) and three individual semi‐structured interviews were conducted with current carers; one focus group (n=4) was conducted with former carers. Individual semi‐structured interviews were offered to family carers who preferred not to participate in a focus group. Four focus groups were conducted with professionals with experience in end of life care in dementia, who were purposively sampled to include a broad range of expertise in dementia end of life care. The practitioner focus groups consisted of between four to eight participants (focus group 1, n=4; focus group 2, n=5; focus group 3, n=8; focus group 4, n=7).

Interviews were conducted by an experienced qualitative researcher (ND), and focus groups were facilitated by a researcher experienced in conducting focus groups (ND, SI, JW), observed by an additional researcher who took field notes (JW, RM).

A topic guide was developed based on findings of preliminary work with family carers of people with dementia^23^, ^24^ and a review of the literature.^25^ The topic guide was used to facilitate the focus group discussions and was modified for use in individual interviews. It was presented to the research development group consisting of practitioners working with people with dementia as well as family carers for feedback and developed further based on these discussions. The same topic guide was used with family carers and professionals, but also explored the family carers’ experiences of caring for a person with dementia within the individual interviews. The guide explored decision making related to the following specific areas:

1. Management of difficulties with swallowing and eating
2. Management of agitation/comfort
3. Ending life sustaining medical treatment
4. Maintaining personhood
5. Providing routine care (ie personal care including washing, changing bed sheets and clothing)
6. Communication between professionals.

Groups were presented with the words of each topic visually using a PowerPoint presentation with a brief explanation from the facilitator and asked to discuss difficult decisions which needed to be made around this topic. This study adopted a ‘think‐aloud’ strategy for the focus groups, which encourages participants to vocalize their thought processes while being presented with a problem or situation.^26^ This method has previously been used to understand difficult decision‐making processes in health care.^27^ Traditional semi‐structured interviews allow people to rationalize their thought processes before speaking and therefore may not provide an understanding of how an individual arrived at their decision.^28^ The results from these interviews highlighted the views and experiences of both family carers and practitioners. The findings formed the basis for the development of a series of heuristics to aid decision making for practitioners caring for people with dementia at the end of life.^29^

Ethics approval for the study was obtained from King's Cross and Camden REC committee (Ref 15/LO/0156). Informed written consent was obtained from all participants prior to conducting the focus groups and interviews. The focus groups lasted between 60 and 80 minutes and individual interviews lasted around one hour.

### 2.4 Analysis

Focus group and individual interviews were audio‐recorded and transcribed verbatim by an external transcriber and checked by (ND). An inductive approach was taken using thematic analysis methods. Each transcript was read independently by two researchers (KL, RM) to familiarize themselves with the data. Following this, line‐by‐line coding was carried out by (KL), who has a background in social sciences and (RM), who is a general practitioner on both family and practitioner transcripts. Coding was discussed between (ND, KL, RM); and as similar and complementary codes were identified in both family carer and practitioner transcripts, a single coding strategy was agreed for both groups. The remainder of the interviews and focus groups were coded using the agreed coding strategy by (KL, RM). After coding of all transcripts and clustering the codes into categories, provisional themes were agreed upon with others in the research team (SI, JW, ND). Themes were revised iteratively, searching for negative and deviant cases to ensure the themes were supported by the data, increasing the rigour of the findings.^30^ Family carer and professional data were analysed together as following initial reading of the transcripts and discussions within the research team, and it was clear there was a high level of overlap between professional and carer views.

## 3 FINDINGS

Four key themes were identified which contributed to making decisions particularly difficult or complicated at the end of life. These include the following: challenges of delivering coherent care in dynamic systems, uncertainty amongst decision makers, internal and external conflict amongst decision makers and a lack of preparedness for the end of life.

### 3.1 Challenges of delivering coherent care in dynamic systems

As dementia progresses, the person with dementia can find themselves moving through the health and social care system. Movement through the care pathway is not linear and is often punctuated by a series of sudden moves between different care environments including hospital admission.

A frequently changing and unfamiliar environment was described as unsettling for both the person with dementia and their family. It can hinder the development of relationships between the person with dementia and those providing their care. Knowing the person well and having a sense of their personal and social identity was said to enable carers and health‐care professionals to make better informed best interests decisions on behalf of a person with dementia. This was thought to be particularly pertinent at the end of life, when the person with dementia may not always able to verbally express themselves. As one carer explained

Really to look at that (best interests), you've got to know what their quality of life was to start with, to be able to judge their loss of quality of life. (C1, Former Carer)

Similar views were held by one of the medical consultants about the value of continuity of relationships:

…the community team who've known this patient for a long time and seen them deteriorate and actually know a bit more about what they would want at the end of their life… (P1, Consultant in Older People Medicine)

As people moved through the care pathway, the lack of familiarity of the person with dementia by health‐care providers inadvertently leads to disease labelling, whereby the individuality and identity of the person is lost and they are defined by their disease. This was considered to be particularly relevant when a person with dementia is admitted to hospital where staff have no information about them. The potential negative impact of disease labelling (diagnostic overshadowing) on decision making was encapsulated by both practitioners and carers:

One thing that concerns me is that people with dementia are lumped together and they're kind of seen as the dementia people. That to me is concerning because then you begin to lose the fact that there's an individual there. (P2, Community Palliative Care Clinical Nurse Specialist)

…they're treating him, you know, like this old person with dementia, not another one, you know, coming in with a UTI or a collapse…sort of getting rid of you as soon as they can and not that sort of personalised care. (C2, Current Carer)

Movement through health and care systems as individuals are passed to new services or services are withdrawn can result in uncertainty about the roles and responsibilities of those involved. At times, decisions may be delayed due to uncertainty about who is best placed to make them, whilst at other times, assumptions may be incorrectly made that discussions around end of life care preferences have already taken place in other settings. This complex dynamic was explained by a practitioner:

There's often ambiguity around who's responsible for end of life decisions…People in hospital always say, well, it should be the GP who knows them really well and the GP may be saying, well, it should be the people in hospital who have specialist expertise and experience. And actually I think the reality is that nobody likes making end of life decisions for people; it's not easy to do, everyone wishes it was someone else's responsibility (P3, Registrar)

As people move between different care settings/environments, there seemed to be a trade‐off between the access to skills and expertise in a different setting vs the benefits of remaining in a familiar place where staff know the person in terms of their journey so far, their routine and preferences. Where a new care environment is considered necessary, family and other regular care workers who have known the person over a period of time can play an important role, supporting professionals in making best interest decisions. They are likely to be able to recognize non‐verbal communication and may be in a better position to understand and respond to the person's needs. The important role of family members in supporting practitioners was recognized by both family members and practitioners:

It is a sustained observation, actually from the family carers that they know little nuances..[which] at times, the ward doctors, the nurses do not. (C3, Former Carer)

*But it also comes back to what we were talking about earlier about talking to the carers or the family because they know the person best… We can't tell you what's wrong with him, but I'm telling you, this is not his normal… we get that day in, day out, don't we?* (P7, Matron)

However, when this resource is not used effectively, because of poor communication or lack of time to involve family, this can complicate decision making further. As one family carer described

…in the past I've been with him to the hospital where they didn't really want…weren't interested in what I was saying. I was saying, this is not normal, but they were just getting on doing their checks and didn't appear to be taking much notice of what I said, really, which I thought was a bit off but, okay, we go along with whatever we get, you know, I thought, okay, that's their way of working. (C4, Current Carer)

There was also a sense of frustration due to the lack of continuity in some settings, where family carers reported often having to retell the same narrative to different health‐care professionals, sometimes, even within the same care setting. One family carer explained

…I don't get to speak to the same person, it's another person and you think, oh, you know, they don't know what I said last time. (C4, Current Carer).

Organisational structures leading to disconnections across settings were reported as making joint working difficult and complicating decision making. Poor or non‐integrated IT systems and a lack of robust procedures for information transfer between settings were identified as exacerbating this disconnection in communication between settings. Such factors were described as resulting in health‐care professionals having to make decisions with incomplete and sometimes inaccurate information:

None of the IT matches up, so there's no shared database or anything of what's gone before, which would be really helpful. Often… people are admitted and we've only got temporary notes for a period of time, so then when things happen in those few days, maybe, or few hours… conversations aren't’ necessarily very clearly documented about things that have happened previously, so then you have to do it again, because you don't know what's been said. It's a real issue. (P4, Palliative care clinical nurse specialist)

Often when people come to us on the unit, we have no information, especially if they come from hospital. Sometimes it's literally like a blank canvas isn't it? And we don't know any habits or triggers or anything. (P6, Ward Sister)

### 3.2 Uncertainty amongst decision makers

Amongst the possible triad of decision makers, the views of the person with dementia are needed, but are most often inaccessible. This means family members or advocates and practitioners must make decisions.

Often decisions were based on the family member's insight about/or knowledge of the values or preferences of the person with dementia. However, they expressed feelings of uncertainty in how to best meet the needs of their relative. Further complications resulted if formal discussion had not taken place or if legal arrangements were not in place. As one carer explained:

It is difficult for me, or anybody else, probably, to understand what's going on in her brain. The only measure I've got is that she is calm, contented and, as far as I'm aware, well looked after… my view would be that she is where she does not want to be…we both were of the view that we didn't want to go that route…My perception is that she is… existing, which is not the situation, when we sort of had our faculties, that we wanted to be in, either of us. In fact, my… I think probably one of the things to bring out is you've got a living will, but it needs to be updated on a regular basis … Now, effectively, that's what I would have put in M's, if we'd done it, because, I mean, she's living on a knife‐edge…(C5, Current Carer)

I mean, she's had breast cancer twice – if something, another cancer developed, my view would be, let it be; keep her comfortable…you think you know what the individual would want. We've been married, what, 50‐odd years, 52 years, but that's my perception. I don't know hers other than what might have been expressed in a living will. (C5, Current Carer)

The often unpredictable nature of decline in dementia adds to the uncertainty. Practitioners felt limited by their ability to explain this unpredictability of decline. There was fear amongst the practitioners of communicating end of life decision making poorly and a fear of getting it wrong thereby losing the trust of those who are relying on their advice and guidance:

We all find it difficult…when we've withdrawn some of the medical treatment, the patient picks up and that's very hard for the relatives to understand and we get them saying, well, we've made a mistake, can you start treating again…(P1 Consultant in Older People Medicine)

Because the problem is, if they do get better that time and go home, and then come back here later, and you have the same conversation…their expectations are completely different, because they (have) heard it before and they got better last time, so why are you not going to treat this time? (P1 Consultant in Older People Medicine)

Differences in skill levels and competence also meant that some practitioners pass on decision‐making tasks to others, as soon as they feel uncertain or are not confident with new clinical or care issues. In some instances, this led to an immediate decision to admit a person to hospital, seemingly resulting from fear and panic. Some hospital staff and clinical specialists felt that insufficient consideration was given to the wider ramifications of initiating a hospital admission:

The relatives and the staff at the homes sometimes panic a bit and the first thing they do is ring an ambulance and the patient's brought in and…they can bounce in and out without us a truly ever saying, well, where's the ceiling of care? Where are we going to stop? (P7, Matron)

I think, especially health care assistants [care home assistants], they're frightened of being accused of not doing the right thing. So this is where we then get into the hospital scenario and it's easier for things to be decided in hospitals, because there are professionals at hand …(P2, Community Palliative Care Clinical Nurse Specialist)

### 3.3 Internal and external conflict amongst decision‐makers

Internal and external conflict amongst individuals can complicate decision making in a number of ways. Internal conflict may occur within individual practitioners whereby their personal values and ethos may differ from what is expected from them in accordance with professional guidelines and regulations. External conflict may occur when a number of individual practitioners who guided by their own individual values and ethos and governed by guidelines and regulations must negotiate the decision‐making process. It can also occur between practitioners and families whose perspectives, values and priorities are likely to differ. Such conflict amongst individuals was described by a practitioner in the following way:

…Culturally….a lot of nurses from different countries have different views around feeding, and around medication at the end of life; so it's quite complex… not only are you dealing with families, you're dealing with clinicians’ views, you're dealing with your colleagues’ views…(P8, Nurse Ward Manager)

A strong driver which appeared to increase internal conflict amongst clinicians was their expectation of themselves to treat the patient and make them better, keeping to a stereotype that the role of the clinician is to prolong life. This was reported to be most common amongst doctors. Some practitioners described the implications of such a situation:

I think it's very easy to make the decision to feed, to treat so they're seen to be doing something and it's not always the right decision for that patient. (P8, Nurse Ward Manager)

In an acute hospital [people] expect interventions and treatment and they don't necessarily expect there to be a lot of multi‐professional discussion before that happens, which is often the case, because it needs to be right for the individual, rather than a reflex, they're not eating, we must whatever happens, get some nutrition in to them. (P4, Palliative care clinical nurse specialist)

In some settings what were described as rigid routines/standard practices, guidelines (rules and regulations) and performance targets limited practitioners’ autonomy and complicated decision making. There was a sense of conflict between working outside guidelines and thereby potentially exposing oneself to potential litigation and complaints vs acting in the best interests of the person with dementia. For example, as one nurse specialist explained, guidelines on how often a person should be turned to prevent pressure sores tended to be followed even it was judged to be causing undue distress for the person with dementia. Ascribing this to fear of the potential legal implications, practitioners said other practitioners (not themselves) were sometimes making decisions to safeguard themselves, rather than what was in the best interests of the person with dementia:

..If people are going to stop doing things like that [following guidelines not in the best interest of the patient], then they need to know that they're going to be supported in that decision…because the other thing people worry about is litigation and complaints. It's all very well, but is there then going to be a complaint from the family that you haven't moved the patient. (P4, Palliative care clinical nurse specialist)

Practitioners were of the view that relatives’ expectations were shaped either by previous experience of dementia amongst people known to them or by occasions where their relatives’ health had declined and then unexpectedly improved (as noted above). This sometimes led to differences of opinion between family carers and practitioners, in terms of what each believes is in the best interests of the person with dementia and can be a potential source of conflict in decision making. Most commonly, examples of such conflict occurred in talking about decision making about what to do if the person with dementia had eating and swallowing difficulties. Practitioners explained that often families would want their relatives to be fed, even if they as clinicians felt this was not appropriate:

[…] it's also about families understanding that, actually, not eating and drinking is often part of the end stage of the dementia process because I don't think a lot of them do understand that. And I know they get lots of information early on in the disease and when the diagnosis is made, but we can't expect them to retain it all at the end and remember they were told this might happen. I think it's just constantly reinforcing that, and also that not every dementia patient behaves the same, because you'll often get somebody say, ‘oh, my Grandad had dementia and he didn't do that’, so you have to explain that there are different patterns, and I think it is time investment and trying to get the carers and the relatives to understand what end‐stage dementia is and that just offering sips and things frequently is as good as putting up drips and NG feeds and we're not actually doing them any favours [using artificial means of nutrition and hydration]. (P1, Consultant in Older People Medicine)

I think there's also the pressure from families, who sometimes lack understanding of the disease process and they've been insisting on the patients being fed, even though they have been made aware of this.(P5, Palliative care nurse)

### 3.4 Lack of preparedness for the end of life

Preparing early for a progressive decline in health and the inevitable end of life phase, when the person with dementia may be unable to convey or express their wishes, was thought to be vital by some participants. Advance care planning was thought to ease the burden of decision making for family and practitioners who may otherwise struggle to make best interest decisions. Practitioners were of the view that there should be more of a joint effort between different organisations and between different care settings to ensure that discussions on advance care planning took place early on when the person was still in a state of ‘well‐being’ to reduce the burden on relatives and not during an acute incident:

…they've come in to us because they're clinically unwell at that time and it shouldn't necessarily be that everything's put on them to say, you know, we're going to stop this because…it's not until they become clinically unwell and then all these conversations to have and it's kind of like, you know, too much sometimes then and that's when you get resentment and sometimes families get quite angry and think you're not giving the treatment. (P9, Senior Ward Nurse)

Practitioners seemed to suggest that most professional dialogue around dementia was centred on the expected cognitive deterioration over time with less recognition that dementia affects physical capabilities, and in particular, that it can be a condition that people die from. Such emphases seemed to lead to some families feeling that they had been ‘caught off guard’ when end of life seemed to be on the horizon that that they were placed in the position of having to make decisions which they felt they were not adequately prepared to make. Timing of discussions is critical but if a person with dementia is not recognized as nearing end of life, then these conversations may not take place. Practitioners acknowledged that there tended to be poor recognition of the dying process even amongst clinicians, which meant that conversations with some families were delayed or absent altogether:

Many distressing situations arose because people weren't identified as dying, so that's something that, as professionals, we need to get better at, is to recognise…not just recognise but to communicating that to family, making sure.. It's not an ambiguous message. (P10, GP liaison)

It's an uncomfortable subject for us; it's also uncomfortable for the relatives and family to have that advance care planning communication, particularly if the patient is currently well. (P1, Consultant in Older People Medicine)

Some reported that often an acute illness or an admission into hospital would have to occur for advance planning discussions to be instigated. And even then, there may still be a revolving door scenario where the person goes in and out of hospital, and advance care planning might never ensue:

I think something we always have trouble with is around, I guess, advance care planning in these situations, so we might get them risk‐managed, we might get them to a point where, when you can get them home, but then they become like frequent flyers, every time they eat they get a chest infection and they will end up back in hospital and we start the process again. (P11, Lead SLT)

A sense of preparedness, understanding and insight into the impact of dementia on the end of life seemed likely to have resulted in a greater level of acceptance amongst some carers, which was said to have a powerful influence on decision making between families and practitioners. This was highlighted through the account of one carer who explained that she had not wanted to put her husband through any unnecessary effort to extend his life:

Well […] the stage that he was at, there was no point in trying to extend life. He, we were at the end. We'd done everything that we could. It was not that we were trying to get rid of him…We had no other, we were only after his best interests.(C3, Former Carer)

## 4 DISCUSSION

### 4.1 Summary

This qualitative study explored factors contributing to difficult decision making for practitioners and family carers of people with dementia at the end of life. Four key themes were identified as follows: the image of a journey of care for a person with dementia, uncertainty amongst decision makers, internal and external conflict amongst decision makers, and a lack of preparedness for the end of life.

### 4.2 Communication

At the end of life, people with dementia are likely to move across care settings. This movement limits the possibility of developing relationships between the triad of potential decision makers (person with dementia, family carer and practitioner), and this is reflected in the findings of the current study. Caron et al. (2005) suggest that quality of the relationship between carers and practitioners is established from the first interaction but that a trusting and supportive relationship between these two parties can take months to years to develop.^14^ This supports our finding that there is likely to be increased difficulty in sharing decision making between staff and family carers who are unfamiliar with each other.

Moves between different settings can limit the knowledge and insight into a person's underlying social and medical history. Having little or no knowledge of the person and not being able to communicate with them can lead to ‘disease labelling’^31^ a situation in which the individuality and identity of the person is lost and they are defined by their disease. Disease labelling can also result in diagnostic overshadowing,^32^ whereby symptoms and signs are incorrectly attributed to dementia and other potential causes overlooked. Some practitioners suggested that use of tools such as ‘This is me’ which have been created to provide medical and social information about the person with dementia, to aid health and social care professionals to see the person as an individual and deliver person‐centred care, should be used more widely^33^

The difficulties of decision making are likely to be even more pronounced at the end of life for people with dementia, compared to other health conditions, because of the associated cognitive decline.^34^, ^35^ Although in our study, those familiar with the person with dementia such as professional care workers or relatives were important in bridging the disconnection through the journey of care, these resources were often not well used. Studies have also highlighted the importance of ‘educating health‐care providers about the importance of working with families, and the importance of investing time in validating this partnership’,^14^ a key component of this work with families is good communication as reported by participants in this present study. Poor interpersonal communication between practitioners in the same setting as well as across settings, and between practitioners and family carers, not surprisingly appeared to increase the difficulty of decision making. Good communication has been consistently highlighted as an important aspect of shared decision making in various health conditions^36^, ^37^, ^38^ as well as in end of life care.^39^ Our study suggests that good communication needs to be considered at system level (eg information sharing) as well as interpersonal levels.

### 4.3 Uncertainty and conflict amongst decision makers

Uncertainty was often a core component of much of the discussion that took place with family carers and practitioners. Not only was uncertainty a potential cause of sudden movement between settings with a direct impact on the person with dementia but it was also likely to create conflict within individuals as well as between the decision makers, indirectly impacting upon the person with dementia. The unpredictable nature of the disease course and at times a lack of recognition of the dying phase appeared to be the main source of uncertainty for practitioners. Other studies have also found that limited understanding, recognition and preparedness of the end of life amongst both practitioners and family members are common.^40^, ^41^

People with dementia can have a decline in health with little expectation of recovery; however, on occasion, there may be a sudden and unexpected improvement. Our findings indicate that the unpredictable nature of the dementia trajectory may lead to practitioners fearing that families will not trust their opinions and decisions. This fear appeared to make communication between carers and practitioners difficult. The theory of cognitive dissonance suggests that individuals strive to ensure that their beliefs and behaviours are consistent. Inconsistency (dissonance) or disharmony results in the decision maker becoming psychologically uncomfortable, motivating them to reduce this dissonance by actively avoiding situations that are likely to increase it.^42^ It may be that this is reflected in our findings amongst some practitioners. The uncertainty might result in avoidance of difficult conversations. This fear also extended to overarching concern about the risk of wider repercussions such as litigation or complaints.

The complexities relating to decision making at end of life amongst those with dementia seemed to leave some practitioners feeling both disarmed and conflicted. Practitioners may feel disarmed because they do not have all the necessary information to make the decision for the person with dementia. In this situation, they must rely on secondary sources for information regarding the preferences of the person with dementia. However, some practitioners appeared to assume that the views and expectations of relatives were shaped by their own previous experience of dementia amongst people known to them. Other practitioners may assume that family carers know what the wishes and preferences of the person with dementia are even when no ACP has been discussed. This may potentially place a great deal of pressure on the relatives as well as lead to situations where decisions are based on what the relatives want as opposed to what the person with dementia may have wanted. Practitioners may feel uneasy because they are likely to be facing a situation in which they have to deal with multiple viewpoints, whilst managing their own perceptions of their role, their values and working within sets of rigid standards and guidelines.

Our study found that some relatives also faced uncertainty and conflict. Their accounts talked mostly about their uncertainty around what their relative would have wanted and feelings that they were unable to do their best for them. Harrison‐Dening and colleagues demonstrated that family carers are not very good at predicting what the person with dementia would have wanted.^43^ Greater preparedness through earlier conversations with the person with dementia and their relatives, ensuring a better understanding of dementia and end of life, as well as advance care planning could be encouraged. Others have also reported limited understanding amongst family carers about what to expect at the end of life.^40^ However, past studies do suggest that even when individuals have made advance directives, such as non‐resuscitation orders, relatives are unsure about how closely to adhere to them.^1^, ^41^ Therefore, it may be important to revisit complex decisions at different time points.

A lack of preparedness can set up a cascade of events, whereby the person with dementia moves between care environments and is exposed to investigations and treatments that are not in line with the goals of care. Once the person with dementia is admitted to hospital, there may be an impetus to do something, in the way of treatment. This may spiral and lead to a situation in which it is more difficult to take a step back and initiate end of life care.

### 4.4 Strengths and limitations

The focus groups carried out in this study included a mix of practitioners from different disciplines. The interdisciplinary mix of the focus groups was valuable to obtain a range of views and perspectives.^44^ Mixed groups have the potential to silence individuals; however, this study found that a mixture of participants encourage conversation, debate and friendly questioning.

There are possible limitations with the sample in particular those who agreed to participate may encompass stronger opinions regarding end of life dementia care and therefore present a potential sample bias. Regional variations in end of life care have been recognized^45^ and participants in our study were all practitioners experienced and working in either dementia or palliative care in London, Greater London, or Essex; therefore, their views may therefore not necessarily be representative of practitioners across England. However, teams did vary in the level of services provided.

Only two focus groups and three interviews were conducted with current and former family carers; it would have been useful to conduct more focus groups and interviews with family carers to allow for an in‐depth comparison of family carer and practitioner views. Family carers were recruited from a national organisation and therefore may provide a biased range of opinions, for example, relating their views to extreme negative or extreme positive experiences they had of caring. Previous research has shown that family carers are more likely to take part in research if they have positive views to report,^46^ however other studies have demonstrated a bias towards those with more negative experiences.^40^

### 4.5 Implications and further work

This study echoes other research conducted on needs and decision making relating to end of life care in dementia.^15^ It suggests that the physical impacts of dementia, beyond cognitive decline, may need to be better recognized by practitioners and that there should be more efforts to engage families in such discussions if they wish. In terms of changes in care settings, decision makers need to consider the impact of moving as weighed against the potential gains. It is likely that some conversations with relatives need to be revisited multiple‐times, as appropriate. Although increased importance is being given to advance care planning, it is evident that the uncertainty around decision making continues; therefore, important conversations between the triad of decision makers need to take place at an early stage. Movement through care settings is likely to complicate decision making and make it unclear as to whether end of life conversations have taken place. The role of GPs may extend to forestalling unnecessary movement through different care settings, facilitating a more seamless journey of care when necessary, and ensuring better transfer of information about the person with dementia. Additionally, there appears to be a pressing need for improvements in informational sharing practices and policy. Practitioners should reflect on their own values and whether the expectations they place on themselves are in line with good decision making for their patients.

Research often concludes within this field that more training is needed for professionals, or that more information is needed for family carers about dementia and end of life in particular. However, we suggest that training is not always enough and guidelines can only guide to some extent. We suggest what is needed is more practical assistance, a tool such as a decision aid that encourages more engagement between professionals and carers, to have difficult conversations and carefully consider difficult decisions which need to be made. A tool such as this may enhance the engagement with advance care planning, and encourage both more professionals and people with dementia and their families to forward plan. Similarly, such a tool may be useful when planning has not taken place and decisions need to be made later on in the course of dementia when the person no longer has capacity. Finally, such a tool could be used as a means of engaging those practitioners and or family in difficult conversation which many so often actively avoid.

## AUTHOR CONTRIBUTIONS

ND, SI, JM & ELS helped develop the project and gain funding; ND, SI, JW, RM, & KL contributed to data collection and analysis. All authors have contributed to drafting this article.

## CONFLICT OF INTEREST

No conflicts of interest have been declared
